# Severity of Nonalcoholic Fatty Liver Disease is Associated With Cardiovascular Outcomes in Patients With Prehypertension or Hypertension: A Community–Based Cohort Study

**DOI:** 10.3389/fendo.2022.942647

**Published:** 2022-08-25

**Authors:** Qi–Rui Song, Shuo–Lin Liu, Qian-Hui Ling, Qian-Nan Gao, Rui-Xue Yang, Shuo-Hua Chen, Shou–Ling Wu, Mu-Lei Chen, Jun Cai

**Affiliations:** ^1^ State Key Laboratory of Cardiovascular Disease of China, Hypertension Center, Fuwai Hospital, National Center for Cardiovascular Diseases of China, Chinese Academy of Medical Sciences and Peking Union Medical College, Beijing, China; ^2^ Department of Cardiology, Zhongshan Hospital, Fudan University, Shanghai Institute of Cardiovascular Diseases, Shanghai, China; ^3^ Department of Cardiology, Kailuan General Hospital, Tangshan, China; ^4^ Heart Center and Beijing Key Laboratory of Hypertension, Department of Cardiology, Beijing Chaoyang Hospital, Capital Medical University, Beijing, China

**Keywords:** nonalcoholic fatty liver disease, prehypertension, hypertension, cardiovascular disease, prognosis

## Abstract

**Background:**

It is unclear whether more severe non–alcoholic fatty liver disease (NAFLD) combined with prehypertension or hypertension is associated with a higher risk of cardiovascular events (CVEs). To evaluate the relationship between the severity of NAFLD and CVEs among patients with prehypertension or hypertension.

**Methods:**

In this prospective community–based Kailuan cohort, participants without cardiovascular disease and alcohol abuse, or other liver diseases were enrolled. NAFLD was diagnosed by abdominal ultrasonography. Prehypertension was defined as systolic blood pressure (BP) of 120–139 mmHg or diastolic BP of 80–89 mmHg. Participants with NAFLD were divided into mild, moderate, and severe subgroups. Follow–up for CVEs including myocardial infarction, hemorrhagic stroke, and ischemic stroke. The Cox proportional hazards model was used to estimate hazard ratios and 95% CIs of CVEs according to the severity of NAFLD and hypertensive statutes. The C-statistic was used to evaluate the efficiency of models.

**Results:**

A total of 71926 participants (mean [SD] age, 51.83 [12.72] years, 53794 [74.79%] men, and 18132 [25.21%] women) were enrolled in this study, 6,045 CVEs occurred during a median of 13.02 (0.65) years of follow–up. Compared with participants without NAFLD, the hazard ratios of CVEs for patients with mild, moderate, and severe NAFLD were 1.143 (95% CI 1.071–1.221, *P* < 0.001), 1.218 (95% CI 1.071–1.221, *P* < 0.001), and 1.367 (95% CI 1.172–1.595, *P* < 0.001), respectively. Moreover, participants with prehypertension plus moderate/severe NAFLD and those with hypertension plus moderate/severe NAFLD had 1.558–fold (95% CI 1.293–1.877, *P* < 0.001) and 2.357–fold (95% CI 2.063–2.691, *P* < 0.001) higher risks of CVEs, respectively, compared with those with normal BP and no NAFLD. Adding a combination of NAFLD and BP status to the crude Cox model increased the C–statistic by 0.0130 (0.0115–0.0158, *P* < 0.001).

**Conclusions:**

Our findings indicated that the increased cardiovascular risk with elevated BP is largely driven by the coexistence of moderate/severe NAFLD, suggesting that the severity of NAFLD may help further stratify patients with prehypertension and hypertension.

## Background

Non–alcoholic fatty liver disease (NAFLD) represents one of the most common causes of chronic liver disease worldwide, with approximately 25% of the general adult population affected by the disease globally (1, 2). The subtype of NAFLD encompasses a spectrum of pathologic conditions ranging from simple steatosis through steatohepatitis to liver fibrosis, cirrhosis and ultimately, hepatic cancer (3). Importantly, accumulating evidence demonstrates that NAFLD is a multifaceted disorder that affects many extrahepatic organ systems, and increases the risks of diabetes mellitus (DM), hypertension, and cardiovascular disease (CVD) (4, 5).

Prehypertension, an intermediate stage between normal blood pressure (BP) and hypertension, is defined as BP in the range of 120–139/80–89 mmHg according to the 2018 Chinese Guidelines for Prevention and Treatment of Hypertension (6). However, controversies remain concerning whether prehypertension is a disease and whether antihypertensive therapy is required in the absence of other CVD (7). Some studies have shown that prehypertension was associated with a higher risk of incident hypertension and CVD events (CVEs) than normal BP of <120/80 mmHg (8, 9), however, conflicting results about this association have also been reported (10–13). Interestingly, a recent study suggested that prehypertension seems to augment the risk of cardiovascular and all-cause mortality when combined with other cardiometabolic disorders (12).

Emerging evidence has revealed that the presence and severity of NAFLD are closely associated with elevated BP and higher prevalence of prehypertension and hypertension (14–16). It has been reported that up to 49.5% of hypertensive individuals have NAFLD, and NAFLD was also independently associated with higher blood pressure in both hypertensive and non–hypertensive participants (15, 17).

Nevertheless, whether NAFLD increases CVD risk in patients with prehypertension and hypertension has never been explored. Herein, this study aimed to investigate the combined effect of NAFLD and prehypertension or hypertension on cardiovascular outcomes in a large Chinese community–based cohort.

## Materials and Methods

### Study Design and Population

We used data from the Kailuan study (trial registration number, ChiCTR-TNC-1100148; trial registration site, http://www.chictr.org.cn/index.aspx), which is an ongoing prospective cohort study performed in Tangshan City, northern China. The detailed information regarding the study design and procedures of the Kailuan study has been published elsewhere (18). Briefly, from June 2006 to October 2007, 101510 participants (81110 men and 20400 women, aged 18–98 years) were recruited in the 11 hospitals affiliated with the Kailuan community. All participants completed questionnaires, physical examinations, and laboratory tests at enrollment, and re-examinations were conducted every 2 years until December 31, 2019, or when death occurred.

**Figure 1:**
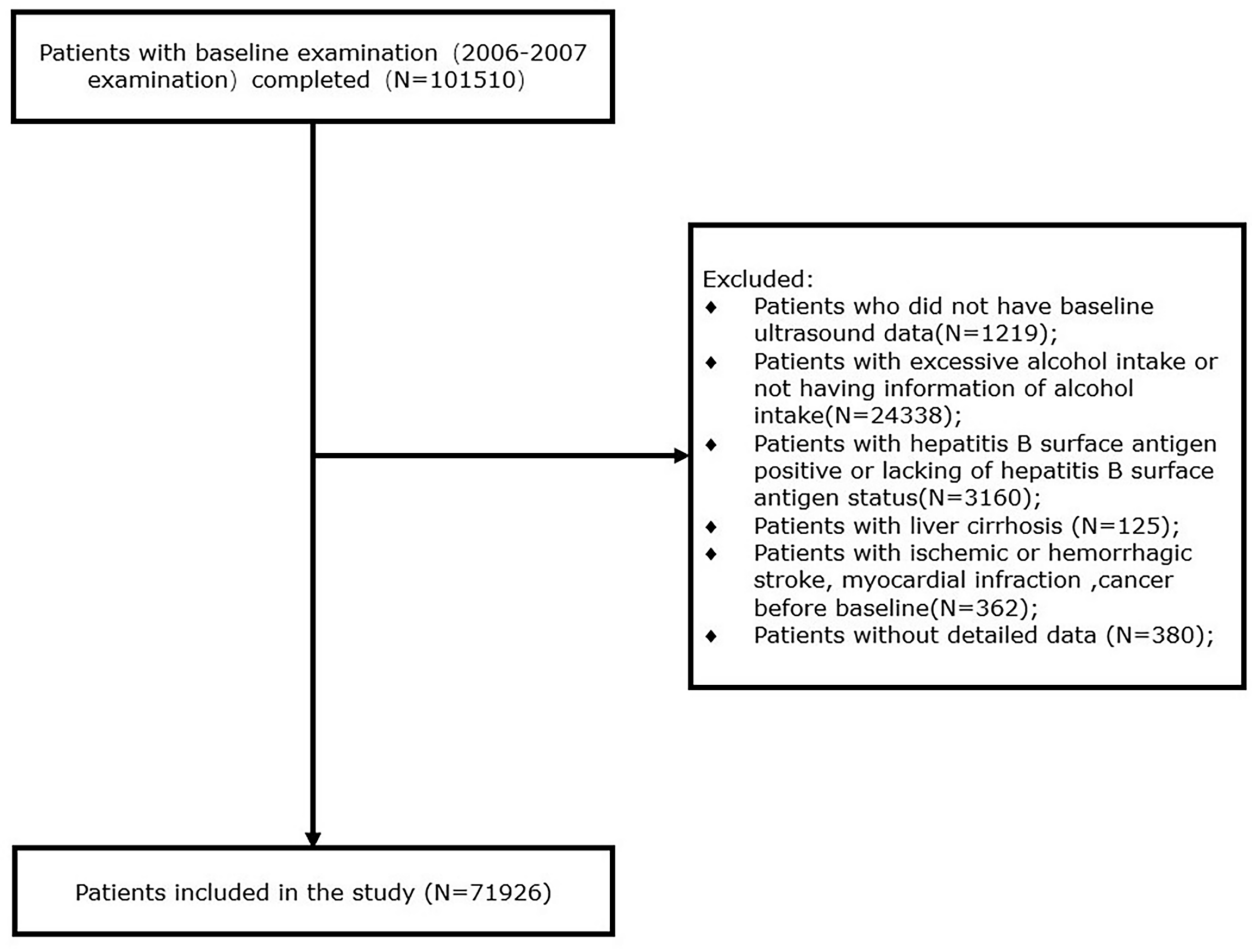
Flowchart of the study.

For the present study, participants who underwent the first survey were included. We excluded 1219 participants who lack baseline ultrasound data, 24338 participants without alcohol intake information or with excessive alcohol consumption (defined as alcohol intake ≥30 g/day for men and ≥20 g/day for women), 3160 participants who had positive hepatitis B surface antigen (HBsAg) or without this information, 125 participants who were diagnosed as liver cirrhosis, 362 participants with events of ischemic or hemorrhagic stroke, myocardial infarction, or cancer before entering the first survey, and 380 participants with missing data on blood pressure. Finally, 71926 participants at baseline were included in this study (**Figure 1**).

This study complied with the Declaration of Helsinki and was approved by the ethics committees of the Kailuan Medical Group, Kailuan Group. All participants have provided their written informed consent.

### Assessment of NAFLD

Based on the criteria for the assessment and management of NAFLD in China (19), NAFLD was diagnosed when participants with at least two of the following abnormal findings on abdominal ultrasonography: (1). increased echogenicity of the liver nearfield region with deep attenuation of the ultrasound signal; (2). hyperechogenicity of liver tissue (“bright liver”), as often compared to hypoechogenicity of the kidney cortex; and (3). Vascular blurring. Participants were diagnosed with NAFLD if they have fatty liver according to abdominal ultrasonography and without excessive alcohol consumption or other causes related to chronic liver diseases (20). As described elsewhere (21), the severity of NAFLD was differentiated by ultrasonography: mild (diffuse increase in fine echoes in liver parenchyma), moderate (diffuse increase in fine echoes with impaired visualization of the intrahepatic vessel borders and diaphragm), and severe (diffuse increase in fine echoes with non-visualization of the intrahepatic vessel borders and diaphragm).

Abdominal ultrasonography was conducted using a high-resolution B-mode topographical ultrasound system with a 3.5 MHz probe (ACUSON X300, Siemens, Germany), and was performed by experienced radiologists who were blinded to clinical presentation and laboratory findings.

### Definition of Hypertension and Prehypertension

BP was measured by well-trained operators between 07:00 and 09:00 at enrollment, and all participants had stayed quietly in a chair for at least five minutes and were prohibited from smoking, caffeine, or physical activity within the 30 minutes before measurement.

BP was measured by a mercury sphygmomanometer, which was regularly checked and calibrated to avoid measurement bias. Systolic blood pressure (SBP) values were recorded when the first of two or more Korotkoff sounds were heard (onset of phase 1), and Diastolic blood pressure (DBP) values were taken as the disappearance of Korotkoff sounds (onset of phase 5). BP measurements were repeated three times at intervals of one to two minutes, and the average value was taken for hypertension diagnosed.

Hypertension is defined as SBP ≥140 mmHg or DBP ≥90mmHg or the self-reported use of antihypertensive treatment according to the 2018 Chinese Guidelines for Prevention and Treatment of Hypertension (6). Prehypertension is defined as SBP between 120 and 139 mmHg or DBP between 80 and 89 mmHg. Normal BP is defined as SBP < 120 mmHg and DBP < 80 mmHg.

### Definition of Outcomes

In the present study, the primary outcomes were the incident CVEs, including myocardial infarction (MI), hemorrhagic stroke (HS), and ischemic stroke (IS). Assessments of CVEs have been described in detail. Briefly, The CVEs were determined from the biennial interviews and each year’s discharge lists which were reviewed and adjudicated by a committee consisting of 3 experienced masked physicians from the 11 Kailuan hospitals, the Municipal Social Insurance Institution which covered all participants in this study. Information on mortality was collected from provincial vital statistics offices and reviewed by clinicians. The CVEs were identified by ICD-10th revision codes. Neurological signs, clinical symptoms, neuroimages (computed tomography or magnetic resonance imaging), and other diagnostic reports, as detailed previously (18) were used for the diagnosis of stroke according to the World Health Organization criteria (22). MI was diagnosed based on clinical symptoms, changes in cardiac enzymes or biomarkers concentrations as well as electrocardiogram, according to the World Health Organization’s Multinational Monitoring of Trends and Determinants in Cardiovascular Disease (MONICA) criteria (23).

### Assessment of Covariates

Information on sociodemographic characteristics including age, sex, smoking status, alcohol consumption, physical activity, education level, and use of lipid-lowering medication was collected by trained interviewers through standard questionnaires. Participants who currently smoke or have a history of smoking were regarded as ever-smokers. Physical activity was defined as engaging in aerobic exercise ≥3 times/week, for ≥30 min/session. Body mass index (BMI) was calculated as weight (kilograms) divided by the squared height (meters).

Blood samples were obtained from the anterior elbow vein of all participants after at least 8 hours of fasting conditions. Concentrations of fasting blood glucose (FBG), High-sensitivity C-reactive protein (Hs-CRP), Alanine aminotransferase (ALT), Total bilirubin (TBIL), creatinine (CR), uric acid (UA), triglyceride (TG), total cholesterol (TC), high-density lipoprotein cholesterol (HDL-C), and low-density lipoprotein cholesterol (LDL-C) were measured using an auto-analyzer (Hitachi 747; Hitachi, Tokyo, Japan) in the central laboratory of Kailuan hospital. The hepatitis B surface antigen (HBsAg) was tested by the enzyme-linked immunosorbent assay (Shanghai Kehua Bio-Engineering, KHB). We calculated the estimated glomerular filtration rate (eGFR) according to the Chronic Kidney Disease Epidemiology Collaboration creatinine equation (24). Hyperlipidemia was defined by the individual’s medical history or fasting total cholesterol (TC) ≥ 5.18 mmol/L or triglyceride (TG)≥1.7 mmol/L.

### Statistical Analysis

We use mean ± standard deviation (SD) or median with an interquartile range (25%–75%) to describe continuous variables, and categorical variables were presented as number (n) or percentage (%). The distributions of variables were examined by Kolmogorov–Smirnov test. We use the One-way ANOVA or Kruskal–Wallis test to compare the baseline variables across the BP status where appropriate, and the Chi-square test was conducted to compare the categorical variables.

We test the event-free survival rates across groups using the Kaplan–Meier method. Univariate and multivariate cox proportional hazard regression models were conducted to calculate the hazard ratios (HR) and 95% confidence intervals (CI) of incident CVEs among groups. In this study, the crude model was unadjusted. Model 1 was adjusted for age, sex, physical activity, BMI (≥30, 25–29.9, 18.5–24.9, <18.5), and smoke. Model 2 was further adjusted for lipid-lowering medications, antidiabetic medications, education level, FBG, HDL, LDL, TG, HsCRP, and eGFR, compared with model 1. Trend tests were conducted in the Cox proportional hazard regression models after the median value of each group was entered into the model and was treated as a continuous variable. To evaluate the efficiency of models and the incremental value of adding the combination of NAFLD and BP status into the original model, the C-statistic and ΔC-statistic were conducted.

The statistical analyses were performed with SAS version 9.4 (SAS Institute, Inc, Cary, NC) and R language, version 3.5.2. All statistical tests were two-sided, with a p-value < 0.05 considered statistically significant.

## Results

### Baseline Characteristics

A total of 71926 participants (mean [SD] age, 51.83 [12.72] years, 53794 [74.79%] men, and 18132 [25.21%] women) were enrolled in this study, those with nonfatty liver, mild NAFLD, moderate NAFLD, and severe NAFLD were 68.64%, 20.29%, 9.10%, and 1.97%, respectively. As presented in **Table 1**, the waist circumference, BMI, systolic blood pressure, diastolic blood pressure, fasting blood glucose, creatinine, TG, LDL–C, HsCRP, and ALT were elevated, while the eGFR was decreased from nonfatty liver to severe NAFLD (all *P* for trend < 0.001, **Table 1**). Besides, the proportion of diabetes, hyperlipidemia, and receiving antihypertensive and antidiabetic medication was higher in patients with NAFLD of different degrees compared with those without NAFLD (*P* for trend < 0.001).

**Table 1:** Clinical characteristics of participants according to the severity of NAFLD.

	Non-Fatty liver (n=49371)	Mild NAFLD (n=14596)	Moderate NAFLD (n=6542)	Severe NAFLD (n=1417)	P value
Age (years)	51.36 ± 13.20	52.88 ± 11.50	52.94 ± 11.57	52.40 ± 11.54	<0.001
Men, n (%)	36421 (73.77)	11268 (77.20)	5042 (77.07)	1063 (75.02)	<0.001
Waist circumference (cm)	84.32 ± 9.39	91.06 ± 8.48	93.62 ± 8.86	96.80 ± 9.59	<0.001
Body mass index, (kg/m^2^)	23.98 ± 3.07	26.92 ± 2.98	28.20 ± 3.23	29.88 ± 3.82	<0.001
Diabetes, n (%)	3234 (6.55)	2169 (14.86)	1291 (19.73)	349 (24.63)	<0.001
Hyperlipidemia, n (%)	19925 (40.36)	9386 (64.31)	4753 (72.65)	1075 (75.86)	<0.001
Physical activity≥3 times/wk, (%)	7212 (14.61)	1937 (13.27)	1000 (15.28)	264 (18.63)	<0.001
Current or previous smoking, n (%)	14344 (29.05)	4584 (31.41)	1858 (28.40)	422 (29.78)	<0.001
Education(college/university), n (%)	3558(4.95)	939(6.43)	376(5.75)	70(4.94)	<0.001
Systolic blood pressure (mmHg)	127.87 ± 20.56	135.58 ± 20.83	139.77 ± 20.87	144.15 ± 21.28	<0.001
Diastolic blood pressure (mmHg)	81.51 ± 11.24	86.19 ± 11.51	88.76 ± 11.87	90.76 ± 11.91	<0.001
Laboratory findings					
Fasting blood glucose, (mmol/L)	5.30 ± 1. 51	5.81 ± 1.99	6.04 ± 2.19	6.24 ± 2.28	<0.001
Creatinine (mg/dL)	91.05 ± 30.08	90.93 ± 26.47	98.71 ± 39.81	102.36 ± 30.82	<0.001
eGFR (ml/min/1.73 m^2^)	81.17 ± 19.51	80.59 ± 19.21	75.46 ± 19.99	71.89 ± 18.75	<0.001
TG (mmol/L)	1.12 (0.81–1.58)	1.64 (1.18–2.45)	1.92 (1.34-2.97)	2.00 (1.41-3.17)	<0.001
TC (mmol/L)	4.86 ± 1.09	5.12 ± 1.16	5.04 ± 1.37	4.99 ± 1.37	<0.001
HDL–C (mmol/L)	1.52 ± 0.35	1.51 ± 0.35	1.49 ± 0.35	1.48 ± 0.37	<0.001
LDL–C (mmol/L)	2.31 ± 0.86	2.34 ± 0.88	2.39 ± 0.86	2.50 ± 1.56	<0.001
HsCRP (mg/L)	0.66 (0.24–1.78)	1.10 (0.43–2.76)	1.30 (0.59-3.10)	1.70 (0.74-3.61)	<0.001
ALT (U/L)	16.00 (12.00–22.00)	20.00 (15.00–27.00)	23.00 (18.00-33.00)	25.00 (19.00-40.00)	<0.001
TBIL (μmol/L)	12.73 ± 4.85	12.86 ± 4.75	12.79 ± 4.94	13.14 ± 4.88	<0.001
Medications					
Antihypertensive medication, n (%)	4206 (8.52)	2350 (16.10)	1237 (18.91)	354 (24.98)	<0.001
Antidiabetic medication, n (%)	985 (2.00)	589 (4.04)	297 (4.54)	103 (7.27)	<0.001
Lipid-lowering medication, n (%)	326 (0.66)	244 (1.67)	131 (2.00)	28 (1.98)	<0.001

eGFR, estimated glomerular filtration rate; TG: triglyceride; TC: total cholesterol; HDL–C, high–density lipoprotein cholesterol; LDL–C: low–density lipoprotein cholesterol; HsCRP, high–sensitivity C–reactive protein; ALT, Alanine aminotransferase; TBIL, total bilirubin.

### Association Between BP Status and Cardiovascular Outcomes

During a median follow–up of 13.02 (0.65) years, 6045 subjects experienced CVEs. The incidences of CVEs in prehypertension and hypertension groups were 5.69% and 12.75%, respectively. Multivariate Cox proportional hazard regression analyses revealed that HRs (95% CIs) for CVEs were 1.191 (95% CI 1.076–1.318) in the prehypertension group and 2.090 (95% CI 1.900–2.300) in the hypertension group compared with normal BP group, after adjusting for potential confounding factors (**Table S2**).

**Figure 2:**
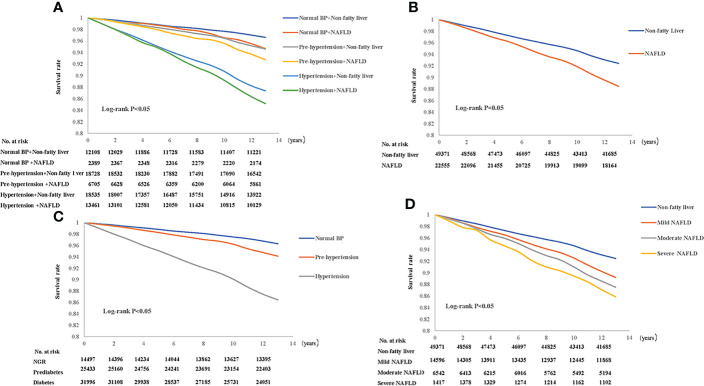
Kaplan–Meier curves by nonalcoholic fatty liver disease (NAFLD) combined with blood pressure (BP) status **(A)**, NAFLD status **(B)**, BP status **(C)**, severity of NAFLD **(D)**.

Besides, Kaplan–Meier analysis with log-rank test revealed that participants with hypertension were least likely to be free of events, while patients with prehypertension had a lower event-free survival rate than the normal BP group (**Figure 2C**, all *P* < 0.05).

### NAFLD, BP Status, and Cardiovascular Outcomes

As presented in **Table S3**, participants with NAFLD had a higher risk of CVD events compared with those with nonfatty liver disease (HR 1.174, 95% CI 1.106–1.246, *P <*0.001), after adjustment for confounding risk factors. Moreover, we further explore the association between the severity of NAFLD (non–NAFLD, mild, moderate, and severe NAFLD) and CVEs. Multivariate Cox regression analysis showed that mild NAFLD, moderate NAFLD, and severe NAFLD had 1.143–fold (95% CI 1.071–1.221, *P* < 0.001), 1.218–fold (95% CI 1.071–1.221, *P* < 0.001), and 1.367–fold (95% CI 1.172–1.595, *P* < 0.001) higher risk of CVEs, respectively, by comparing with nonfatty liver group (**Table S3**).

Furthermore, patients were grouped according to both BP status and the severity of NAFLD. Multivariate Cox proportional hazard regression model demonstrated that compared with patients in normal BP and nonfatty liver group, patients in prehypertension plus mild NAFLD and hypertension plus mild NAFLD groups had 1.251–fold (95% CI 1.072–1.458), and 2.304–fold (95% CI 2.039–2.603) higher risk of CVEs (all *P* < 0.001, **Table 2**). Moreover, participants with moderate/severe NAFLD were associated with 1.558–fold (95% CI 1.293–1.877) and 2.357–fold (95% CI 2.063–2.691) higher risk of CVEs in prehypertension and hypertension groups, respectively.

**Table 2:** The severity of nonalcoholic fatty liver disease in relation to cardiovascular outcomes in patients with different BP status.

	Events/subjects (6045/71926)	HR (95% CI)					
		Crude model	P value	Model 1	P value	Model 2	P value
Normal blood pressure							
Nonfatty liver	(399/12108)	Reference		Reference		Reference	
Mild NAFLD	(89/1801)	1.560 (1.234–1.972)	<0.001	1.299 (1.026–1.645)	0.030	1.129 (0895–1.423)	0.305
Moderate/Severe NAFLD	(32/588)	1.601 (1.097–2.368)	0.015	1.309 (0.895–1.913)	0.165	1.177 (0.819–1.423)	0.379
Prehypertension							
Nonfatty liver	(977/18728)	1.625 (1.441–1.833)	<0.001	1.247 (1.105–1.408)	<0.001	1.189 (1.057–1.337)	0.004
Mild NAFLD	(302/4670)	2.073 (1.779–2.417)	<0.001	1.487 (1.272–1.738)	<0.001	1.251 (1.072–1.458)	0.004
Moderate/Severe NAFLD	(168/2035)	2.654 (2.207–3.192)	<0.001	1.931 (1.599–2.332)	<0.001	1.558 (1.293–1.877)	<0.001
Hypertension							
Nonfatty liver	(2192/18535)	3.901 (3.493–4.357)	<0.001	2.243 (2.002–2.513)	<0.001	2.086 (1.869–2.330)	<0.001
Mild NAFLD	(1114/8125)	4.580 (4.069–5.155)	<0.001	2.761 (2.439–3.126)	<0.001	2.304 (2.039–2.603)	<0.001
Moderate/Severe NAFLD	(772/5336)	4.837 (4.268–5.482)	<0.001	2.942 (2.574–3.363)	<0.001	2.357 (2.063–2.691)	<0.001
P for trend		<0.001		<0.001		<0.001	

HR, hazard ratio; NAFLD, non-alcoholic fatty liver disease; BMI, body mass index; FBG, fasting blood glucose; HDL, high-density lipoprotein; LDL, low-density lipoprotein; HsCRP, high-sensitivity C-reactive protein; TG, triglyceride; eGFR, estimated glomerular filtration rate. Model 1 was adjusted for age, sex, physical activity, BMI (≥30, 25–29.9, 18.5–24.9, <18.5), smoke. Model 2 was adjusted for age, sex, physical activity, education, BMI (≥30, 25–29.9, 18.5–24.9, <18.5), smoke, FBG, antidiabetic medication, lipid-lowering medication, TG, LDL, HDL, HsCRP and eGFR.

Kaplan–Meier analysis indicated that patients with NAFLD had higher risk of CVEs (**Figure 2B**) than those who without NAFLD. And patients with more severe NAFLD had significantly higher risk of CVEs (**Figure 2D**). Meanwhile, prehypertension plus NAFLD and hypertension plus NAFLD had significantly lower event-free survival rates relative to the reference group (nonfatty liver and normal BP group) (**Figure 2A**) (all *P* < 0.05).

In addition, we further analyzed the relationship between NAFLD combined with hypertension status and all-cause death. After adjusting for confounders, the Cox regression analysis showed there was no significant association between NAFLD status and higher risk of all-cause death when compared with the nonfatty liver group (**Table S5**). The multivariate Cox model demonstrated that when compared with patients in normal BP and nonfatty liver group (**Table S7**), participants with mild NAFLD and moderate/severe NAFLD were associated with 1.516 –fold (95% CI 1.376–1.670) and 1.544–fold (95% CI 1.387–1.720) higher risk of all-cause death in hypertension group.

### Incremental Value of BP Status and NAFLD Over Conventional Risk Factors for CVEs

In Cox prediction model of traditional risk factors, C–statistic value was 0.7228 (95% CI 0.7171–0.7285, **Table 3**). Addition of the severity of NAFLD to the original model improved the C–statistic to 0.7232 [△C–statistic 0.0004 (0.0002–0.0007), *P* < 0.001]. Similarly, adding the BP status to the original model increased the C–statistic to 0.7356 (0.7300–0.7413) [△C–statistic 0.0129 (0.0113–0.0158), *P* = 0.0023]. Finally, when adding both the severity of NAFLD and BP status into the original model, the C–statistic significantly increased to 0.7358 (0.7301–0.7414) [△C–statistic 0.0130 (0.0115–0.0158), *P* < 0.001].

**Table 3:** Incremental predictive values of nonalcoholic fatty liver disease and BP status for cardiovascular outcomes.

Models	C–statistic (95% CI)	ΔC–statistic (95% CI)	P value
original model	0.7228 (0.7171–0.7285)	–	–
original model + severity of NAFLD	0.7232 (0.7174–0.7289)	0.00039 (0.00023–0.00074)	0.0023
original model + BP status	0.7356 (0.7300–0.7413)	0.01285 (0.01128–0.01567)	<0.001
original model + severity of NAFLD+ BP status	0.7358 (0.7301–0.7414)	0.01298 (0.01146–0.01581)	<0.001

NAFLD, non-alcoholic fatty liver disease; BMI, body mass index; FBG, fasting blood glucose; HDL, high-density lipoprotein; LDL, low-density lipoprotein; HsCRP, high-sensitivity C-reactive protein; TG, triglyceride; eGFR, estimated glomerular filtration rate. The original model included age, sex, physical activity, BMI (≥30, 25–29.9, 18.5–24.9, <18.5), smoke, FBG, antidiabetic medication, lipid-lowering medication, TG, LDL, HDL, HsCRP, and eGFR.

## Discussion

In this community–based study of 71926 participants, we showed that higher severity of NAFLD was associated with increased CVE risk. Moreover, patients with prehypertension and hypertension had a 1.191–fold and 2.090–fold increased risk of CVEs compared with those with normal BP. Besides, when patients were stratified by both the severity of NAFLD and BP status, the prehypertension patients who also had more severe NAFLD had a worse prognosis. Furthermore, those with hypertension and severe NAFLD were with the highest CVE risk. Importantly, incorporating the severity of NAFLD into the traditional risk prediction model improved cardiovascular risk prediction, while the incorporation of both NAFLD and BP status into the traditional model does further improve the predictive value. Overall, the present study revealed that patients with prehypertension and severe NAFLD tend to have a worse cardiovascular prognosis.

NAFLD, the most common chronic liver disease, is tightly linked to insulin resistance (IR), obesity, metabolic syndrome, diabetes, and hypertension (1). Apart from liver-related complications, NAFLD causes considerable extrahepatic morbidity and mortality, especially in cardiovascular events (25–27). Furthermore, strong evidence demonstrated that higher severity of NAFLD is associated with a greater risk of CVEs and other cardiac complications, comprising aortic valve calcification, arrhythmias, and left ventricular (LV) hypertrophy (25). A recent Korean nationwide cohort study including 9584399 adult individuals showed that patients with NAFLD had significantly higher CVE risk during a median period of 10.1 years (28). Similarly, in a meta-analysis of 34043 participants over a median follow–up time of 6.9 years, NAFLD is associated with an increased risk of fatal and non–fatal CVEs (26). Besides, patients with more severe NAFLD also have higher incidence of fatal and non–fatal CVEs (27).

BP in the prehypertension range is associated with higher risk of incident hypertension and CVEs than normal BP (7). It has been demonstrated that prehypertension was associated with increased LV mass index, wall thickness, increased incidence of abnormal LV geometry, and impaired diastolic function, suggesting mildly elevated BP was associated with abnormalities of cardiac structure and function (29). Moreover, epidemiologic studies reported that approximately one–third of CVEs in the general population that attributable to elevated BP alone were seemed to occur in individuals with prehypertension (30, 31). A previous prospective cohort study including 53163 healthy adults showed that prehypertension was not associated with an increased risk of cardiovascular mortality during a follow–up period of 5.7 years (32). Nevertheless, some large–scale cohort studies revealed that prehypertension significantly increased the CVD risk (13, 31, 33, 34). For example, the Linxian General Population Trial Cohort enrolled 29439 participants and showed that patients with prehypertension had a higher risk of all-cause mortality, cardiovascular disease, and stroke mortality during a followed-up period for 30 years (31). A meta-analysis including 1129098 participants showed that prehypertension was associated with increased risk of total CVD (RR 1.28, 95% CI 1.16–1.40), coronary heart disease (RR 1.12, 95% CI 1.02–1.23), and stroke mortality (RR 1.41, 95% CI 1.28–1.56) (13). The present study also found that prehypertension was associated with a 1.207–fold increased risk of CVEs compared with those with normal BP. The potential pathophysiologic mechanisms concerning prehypertension and increased cardiovascular risk may attribute to higher cyclic stress on the artery walls, which leads to impaired integrity of the endothelial cell layer and arterial stiffness, consequently resulting in the higher incidence of CVEs.

Notably, NAFLD and hypertension have many common pathophysiologic mechanisms, such as systemic inflammation response (primarily mediated by activated innate immune system and proinflammatory chemokines) and elevated oxidative stress, which may accelerate the progression of circulatory vascular injury (35). On the one hand, hypertension has been associated with advanced fibrosis progression of NAFLD (36). It has been demonstrated, in animal models, the activity of renin-angiotensin-aldosterone (RASS) affects both the progress of hypertension and liver fibrosis (5). Furthermore, polymorphisms of specific gene encoding angiotensinogen are more frequent in patients with NASH than in control individuals, and angiotensin II type 1 receptor polymorphisms is associated with the occurrence of NASH (5, 37). Antagonizing the RASS system could effectively delay the development of liver fibrosis in patients with NAFLD (38). On the other hand, insulin resistance (IR), chronic inflammation, and reactive oxygen species production in NAFLD may accelerate the progression of prehypertension and hypertension, possibly by inducing the expression of inflammatory cytokines and activation of the sympathetic nervous system and RAAS (35). Specifically, IR and dyslipidemia trigged by NAFLD are also closely associated with endothelial dysfunction and vasoconstriction by decreasing endothelial nitric oxide production and activating the mitogen-activated protein kinase pathway (39).

Moreover, accumulating evidence reported a robust association between the presence and severity of NAFLD and the presence of both the prehypertension and hypertension (5, 40, 41). Not only increased BP levels, even mildly elevated BP, is associated with more progressive NAFLD, but the presence of NAFLD identified by ultrasonography or hepatic enzyme levels could predict higher risk of incidence of hypertension (42, 43). The Framingham Heart Study indicated that individuals with hypertension or other metabolic disorders at baseline all had higher odds of incident NAFLD over a follow–up 6 years (42). Notably, another study from China recruited 27769 with BP in the normal range showed that HRs for NAFLD were significantly increased with elevated BP levels (44).

Similarly, studies with the NAFLD diagnosed by computed tomography or ultrasonography have reported that NAFLD also confers a higher risk of hypertension. Two prospective studies revealed that more progressive NAFLD is independently associated with the progression of prehypertension and hypertension (45, 46). Thus, the studies mentioned above demonstrated a reciprocal relationship between NAFLD and hypertension, and they make cause and effect with each other (42, 47). More importantly, whether NAFLD plus prehypertension or hypertension confers a worse cardiovascular outcome remains unknown. Our study found that patients with moderate/severe NAFLD and prehypertension and hypertension are at 1.558–fold and 2.357–fold higher risks of CVEs after adjustment for potential confounding factors. The severity of NAFLD and hypertensive statutes provide additional value for cardiovascular risk prediction in the community-based population.

This study has several limitations. Firstly, as the inherent nature of the observational study, the confounding factors across this study might cause an overestimation of the relationship. We did not examine the hepatitis C virus or other factors that may affect the liver function, the information on cTnT(I) and BNP (NT-proBNP) which may affect the cardiac function was not collected in this cohort. Secondly, NAFLD was only diagnosed by liver ultrasonographic scan and we could not carry out liver biopsy, which might attenuate the diagnostic accuracy of the severity of NAFLD. Finally, the population of this study came from a community in China, which may restrict the applicability and generalizability to other ethnicities.

## Conclusions

In this population-based prospective study, we have shown that more severe NAFLD was an independent risk factor for CVEs. Furthermore, the presence of moderate/severe NAFLD and prehypertension or hypertension was associated with worse cardiovascular prognosis, suggesting that the severity of NAFLD may provide additional value in risk prediction for patients with prehypertension or hypertension.

## Data Availability Statement

The raw data supporting the conclusions of this article will be made available by the authors, without undue reservation.

## Ethics Statement

The studies involving human participants were reviewed and approved by the ethics committees of the Kailuan Medical Group. The patients/participants provided their written informed consent to participate in this study.

## Author Contributions

JC, MC and S-LW planned the study, Q-RS and S-LL conducted a survey, Q-RS and S-LL analyzed the data and wrote the article. Q-RS, S-LL, Q-NG, Q-HL, R-XY and S-HC contributed to the drafting. All authors read and approved the final manuscript. All authors contributed to the article and approved the submitted version.

## Funding

This work was supported by CAMS Innovation Fund for Medical Sciences (No. 2021-1-I2M-007), National Natural Science Foundation of China (No. 81825002, No. 81800367), Beijing Outstanding Young Scientist Program (No. BJJWZYJH01201910023029), Capital Clinical Diagnosis and Treatment Technology Research and Demonstration Application Project of Beijing Science and Technology Commission (No. Z191100006619106), AI+ Health Collaborative Innovation Cultivation Project of Beijing Science and Technology Commission (No. Z201100005620006).

## Acknowledgments

We thanked patient advisers for the information they provided.

## Conflict of Interest

The authors declare that the research was conducted in the absence of any commercial or financial relationships that could be construed as a potential conflict of interest.

The reviewer YZ declared a shared affiliation, with no collaboration, with the authors, to the handling editor at the time of the review.

## Publisher’s Note

All claims expressed in this article are solely those of the authors and do not necessarily represent those of their affiliated organizations, or those of the publisher, the editors and the reviewers. Any product that may be evaluated in this article, or claim that may be made by its manufacturer, is not guaranteed or endorsed by the publisher.

## Abbreviations

CVEs: cardiovascular events; HR, hazard ratio; NAFLD, non-alcoholic fatty liver disease; BMI, body mass index; FBG, fasting blood glucose; HDL, high-density lipoprotein; LDL, low-density lipoprotein; HsCRP, high-sensitivity C-reactive protein; TG, triglyceride; eGFR, estimated glomerular filtration rate.
