# Supplementary material for: Severity of Nonalcoholic Fatty Liver Disease is Associated With Cardiovascular Outcomes in Patients With Prehypertension or Hypertension: A Community–Based Cohort Study
Source: Front Endocrinol (Lausanne). 2022 Aug 25;13:942647. doi: 10.3389/fendo.2022.942647 (PMC9453754; doi:10.3389/fendo.2022.942647)
Supplement: Supplementary file 1 [file DataSheet_1.docx]

**Supplementary materials**

**Table S1.** Clinical characteristics of participants according to NAFLD status

|  | **Total**  **(n= 71926)** | **Non-Fatty liver**  **(n=49371)** | **NAFLD**  **(n=22555)** | **P value** |
| --- | --- | --- | --- | --- |
| Age (years) | 51.83±12.72 | 51.36±13.20 | 52.86±11. 52 | <0.001 |
| Men, n (%) | 53794 (74.79) | 36421 (73.77) | 17373 (77.03) | <0.001 |
| Waist circumference (cm) | 86.78±9.91 | 84.32±9.39 | 92.16±8.82 | <0.001 |
| Body mass index, (kg/m^2^) | 25.07±3.52 | 23.98±3.07 | 27.48±3.23 | <0.001 |
| Diabetes, n (%) | 7043 (9.79) | 3234 (6.55) | 3809(16.89) | <0.001 |
| Hyperlipidemia, n (%) | 35139 (48.85) | 19925 (40.36) | 15314 (67.90) | <0.001 |
| Physical activity≥3 times/wk, (%) | 10413 (14.48) | 7212 (14.61) | 3201 (14.19) | <0.001 |
| Current or previous smoking, n (%) | 21208 (29.49) | 14344 (29.05) | 6864 (30.43) | <0.001 |
| Education(college/university), n (%) | 4943(6.87) | 3558(4.95) | 1385(1.93) | <0.001 |
| Systolic blood pressure (mmHg) | 130.83±21.17 | 127.87±20.56 | 137.33±21.02 | <0.001 |
| Diastolic blood pressure (mmHg) | 83.30±11.70 | 81.51±11.24 | 87.22±11.73 | <0.001 |
| Laboratory findings |  |  |  |  |
| Fasting blood glucose, (mmol/L) | 5.49±1.73 | 5.30±1. 51 | 5.90±2.07 | <0.001 |
| Creatinine (mg/dL) | 91.94±30.54 | 91.05±30.08 | 93.90±31.46 | <0.001 |
| eGFR (ml/min/1.73 m^2^) | 80.35±19.58 | 81.17±19.51 | 78.56±19.62 | <0.001 |
| TG (mmol/L) | 1.27 (0.90–1.91) | 1.12 (0.81–1.58) | 1.75 (1.23–2.62) | <0.001 |
| TC (mmol/L) | 4.93±1.14 | 4.86±1.09 | 5.09±1.24 | <0.001 |
| HDL–C (mmol/L) | 1.52±0.35 | 1.52±0.35 | 1.50±0.35 | <0.001 |
| LDL–C (mmol/L) | 2.33±0.87 | 2.31±0.86 | 2.37±0.87 | <0.001 |
| HsCRP (mg/L) | 0.80 (0.30–2.11) | 0.66 (0.24–1.78) | 1.20 (0.50–2.92) | <0.001 |
| ALT (U/L) | 18.00 (13.00–24.00) | 16.00 (12.00–22.00) | 21.00 (16.00–29.00) | <0.001 |
| TBIL (μmol/L) | 12.77±4.84 | 12.73±4.85 | 12.86±4.82 | <0.001 |
| Medications |  |  |  |  |
| Antihypertensive medication, n (%) | 8147 (11.33) | 4206 (8.52) | 3941 (17.47) | <0.001 |
| Antidiabetic medication, n (%) | 1974 (2.74) | 985 (2.00) | 989 (4.38) | <0.001 |
| Lipid–lowering medication, n (%) | 729 (1.01) | 326 (0.66) | 403 (1.79) | <0.001 |

Abbreviations: eGFR, estimated glomerular filtration rate; TG: triglyceride; TC: total cholesterol; HDL–C, high–density lipoprotein cholesterol; LDL–C: low–density lipoprotein cholesterol; HsCRP, high–sensitivity C–reactive protein; ALT, Alanine aminotransferase; TBIL, total bilirubin.

**Table S2.** Relation of the different blood pressure (BP) status and cardiovascular outcomes in univariate and multivariate survival analysis.

|  | **Events/subjects (****6045/71926)** | **HR (95% CI)** | | | | | |
| --- | --- | --- | --- | --- | --- | --- | --- |
|  |  | **Crude model** | **P value** | **model 1** | **P value** | **model 2** | **P value** |
| **Normal BP** | (520/14497) | Reference |  | Reference |  | Reference |  |
| **Pre–hypertension** | (1447/25433) | 1.614 (1.460–1.784) | <0.001 | 1.241 (1.122–1.373) | <0.001 | 1.191 (1.076–1.318) | <0.001 |
| **Hypertension** | (4078/31996) | 3.913 (3.571–4.287) | <0.001 | 2.290 (2.083–2.517) | <0.001 | 2.090 (1.900–2.300) | <0.001 |

Abbreviations: HR, hazard ratio; NAFLD, non-alcoholic fatty liver disease; BMI, body mass index; FBG, fasting blood glucose; HDL, high-density lipoprotein; LDL, low-density lipoprotein; HsCRP, high-sensitivity C-reactive protein; TG, triglyceride; eGFR, estimated glomerular filtration rate. Model 1 was adjusted for age, sex, physical activity, BMI (≥30, 25–29.9, 18.5–24.9, <18.5), smoke. Model 2 was adjusted for age, sex, physical activity, education, BMI (≥30, 25–29.9, 18.5–24.9, <18.5), smoke, FBG, antidiabetic medication, lipid-lowering medication, TG, LDL, HDL, HsCRP and eGFR.

**Table S3.** Relation of the Nonalcoholic fatty liver disease and cardiovascular outcomes in univariate and multivariate survival analysis.

| **Variables** | **Events/subjects (6045/71926)** | **HR (95% CI)** | | | | | |
| --- | --- | --- | --- | --- | --- | --- | --- |
|  |  | **Crude model** | **P value** | **model 1** | **P value** | **model 2** | **P value** |
| **The presence of NAFLD** | | | | | | | |
| Nonfatty liver | (3568/49371) | Reference |  | Reference |  | Reference |  |
| NAFLD | (2477/22555) | 1.553 (1.475–1.634) | <0.001 | 1.344 (1.269–1.423) | <0.001 | 1.174 (1.106–1.246) | <0.001 |
| **The severity of NAFLD** | | | | | | | |
| Nonfatty liver | (3568/49371) | Reference |  | Reference |  | Reference |  |
| Mild NAFLD | (1501/14596) | 1.452 (1.367–1.542) | <0.001 | 1.282 (1.203–1.367) | <0.001 | 1.143 (1.071–1.221) | <0.001 |
| Moderate NAFLD | (781/6542) | 1.694 (1.568–1.831) | <0.001 | 1.441 (1.326–1.567) | <0.001 | 1.218 (1.071–1.221) | <0.001 |
| Severe NAFLD | (191/1417) | 1.953 (1.688–2.259) | <0.001 | 1.672 (1.436–1.948) | <0.001 | 1.367 (1.172–1.595) | <0.001 |

Abbreviations: HR, hazard ratio; NAFLD, non-alcoholic fatty liver disease; BMI, body mass index; FBG, fasting blood glucose; HDL, high-density lipoprotein; LDL, low-density lipoprotein; HsCRP, high-sensitivity C-reactive protein; TG, triglyceride; eGFR, estimated glomerular filtration rate. Model 1 was adjusted for age, sex, physical activity, BMI (≥30, 25–29.9, 18.5–24.9, <18.5), smoke. Model 2 was adjusted for age, sex, physical activity, education, BMI (≥30, 25–29.9, 18.5–24.9, <18.5), smoke, FBG, antidiabetic medication, lipid-lowering medication, TG, LDL, HDL, HsCRP and eGFR.

**Table S4.** Nonalcoholic fatty liver disease in relation to cardiovascular outcomes in patients with different BP status

|  |  | **HR (95% CI)** | | | | | |
| --- | --- | --- | --- | --- | --- | --- | --- |
| **Variables** | **Events/subjects (6045/71926)** | **Crude model** | **P value** | **model 1** | **P value** | **model 2** | **P value** |
| **Normal BP** |  | | | | | | |
| Nonfatty liver | (399/12108) | Reference |  | Reference |  | Reference |  |
| NAFLD | (121/2389) | 1.570 (1.274–1.935) | <0.001 | 1.298 (1.051–1.603) | 0.015 | 1.138 (0.926–1.398) | 0.219 |
| **Pre–hypertension** |  | | | | | | |
| Nonfatty liver | (977/18728) | 1.625 (1.441–1.833) | <0.001 | 1.247 (1.105–1.407) | <0.001 | 1.188 (1.056–1.336) | 0.004 |
| NAFLD | (470/6705) | 2.249 (1.961–2.579) | <0.001 | 1.614 (1.402–1.858) | <0.001 | 1.341 (1.167–1.541) | <0.001 |
| **Hypertension** |  | | | | | | |
| Nonfatty liver | (2192/18535) | 3.901 (3.493–4.357) | <0.001 | 2.241(2.000–2.510) | <0.001 | 2.084 (1.866–2.327) | <0.001 |
| NAFLD | (1886/13461) | 4.682 (4.187–5.235) | <0.001 | 2.818 (2.502–3.174) | <0.001 | 2.314 (2.057–2.603) | <0.001 |
| **P for trend** |  | <0.001 | | <0.001 | | <0.001 | |

Abbreviations: HR, hazard ratio; NAFLD, non-alcoholic fatty liver disease; BMI, body mass index; FBG, fasting blood glucose; HDL, high-density lipoprotein; LDL, low-density lipoprotein; HsCRP, high-sensitivity C-reactive protein; TG, triglyceride; eGFR, estimated glomerular filtration rate. Model 1 was adjusted for age, sex, physical activity, BMI (≥30, 25–29.9, 18.5–24.9, <18.5), smoke. Model 2 was adjusted for age, sex, physical activity, education, BMI (≥30, 25–29.9, 18.5–24.9, <18.5), smoke, FBG, antidiabetic medication, lipid-lowering medication, TG, LDL, HDL, HsCRP and eGFR.

**Table S5.** Relation of the Nonalcoholic fatty liver disease and all cause death in univariate and multivariate survival analysis.

| **Variables** | **HR (95% CI)** | | | | | |
| --- | --- | --- | --- | --- | --- | --- |
|  | **Crude model** | **P value** | **model 1** | **P value** | **model 2** | **P value** |
| **The presence of NAFLD** |  |  |  |  |  |  |
| Nonfatty liver | Reference |  | Reference |  | Reference |  |
| NAFLD | 1.126 (1.079–1.176) | <0.001 | 1.135 (1.082–1.191) | <0.001 | 1.029(0.978–1.018) | 0.271 |
| **The severity of NAFLD** |  |  |  |  |  |  |
| Nonfatty liver | Reference |  | Reference |  | Reference |  |
| Mild NAFLD | 1.091 (1.036–1.148) | <0.001 | 1.110 (1.051–1.171) | <0.001 | 1.023 (0.968–1.082) | 0.413 |
| Moderate NAFLD | 1.182 (1.104–1.266) | <0.001 | 1.169 (1.086–1.258) | <0.001 | 1.024 (0.950–1.105) | 0.534 |
| Severe NAFLD | 1.237 (1.079–1.418) | 0.002 | 1.326 (1.150–1.528) | <0.001 | 1.132 (0.981–1.306) | 0.09 |

Abbreviations: HR, hazard ratio; NAFLD, non-alcoholic fatty liver disease; BMI, body mass index; FBG, fasting blood glucose; HDL, high-density lipoprotein; LDL, low-density lipoprotein; HsCRP, high-sensitivity C-reactive protein; TG, triglyceride; eGFR, estimated glomerular filtration rate. Model 1 was adjusted for age, sex, physical activity, BMI (≥30, 25–29.9, 18.5–24.9, <18.5), smoke. Model 2 was adjusted for age, sex, physical activity, education, BMI (≥30, 25–29.9, 18.5–24.9, <18.5), smoke, FBG, antidiabetic medication, lipid-lowering medication, TG, LDL, HDL, HsCRP and eGFR.

**Table S6.** Nonalcoholic fatty liver disease in relation to all cause death in patients with different BP status

|  | **HR (95% CI)** | | | | | |
| --- | --- | --- | --- | --- | --- | --- |
| **Variables** | **Crude model** | **P value** | **model 1** | **P value** | **model 2** | **P value** |
| **Normal BP** | | | | | | |
| Nonfatty liver | Reference |  | Reference |  | Reference |  |
| NAFLD | 1.117 (0.936–1.332) | 0.2187 | 1.075 (0.900–1.284) | 0.423 | 0.993 (0.831–1.188) | 0.942 |
| **Pre -hypertension** | | | | | | |
| Nonfatty liver | 1.664 (1.523–1.818) | <0.001 | 1.128 (1.032–1.233) | 0.008 | 1.113 (1.018–1.217) | 0.02 |
| NAFLD | 1.587 (1.422–1.771) | <0.001 | 1.165 (1.041–1.303) | 0.008 | 1.059 (0.945–1.187) | 0.3204 |
| **Hypertension** | | | | | | |
| Nonfatty liver | 3.733 (3.440–4.051) | <0.001 | 1.579 (1.452–1.716) | <0.001 | 1.501 (1.381–1.633) | <0.001 |
| NAFLD | 3.282 (3.015–3.574) | <0.001 | 1.753 (1.601–1.919) | <0.001 | 1.528 (1.393–1.676) | <0.001 |

Abbreviations: HR, hazard ratio; NAFLD, non-alcoholic fatty liver disease; BMI, body mass index; FBG, fasting blood glucose; HDL, high-density lipoprotein; LDL, low-density lipoprotein; HsCRP, high-sensitivity C-reactive protein; TG, triglyceride; eGFR, estimated glomerular filtration rate. Model 1 was adjusted for age, sex, physical activity, BMI (≥30, 25–29.9, 18.5–24.9, <18.5), smoke. Model 2 was adjusted for age, sex, physical activity, education, BMI (≥30, 25–29.9, 18.5–24.9, <18.5), smoke, FBG, antidiabetic medication, lipid-lowering medication, TG, LDL, HDL, HsCRP and eGFR.

**Table S7.** Severity of nonalcoholic fatty liver disease in relation to c all cause death in patients with different BP status.

|  | **HR (95% CI)** | | | | | |
| --- | --- | --- | --- | --- | --- | --- |
|  | **Crude model** | **P value** | **Model 1** | **P value** | **Model 2** | **P value** |
| **Normal BP** | | | | | | |
| Nonfatty liver | Reference |  | Reference |  | Reference |  |
| Mild NAFLD | 1.212 (1.000–1.468) | 0.05 | 1.116 (0.961–1.414) | 0.12 | 1.086 (0894–1.319) | 0.40 |
| Moderate/Severe NAFLD | 0.832 (0.570–1.213) | 0.339 | 0.806 (0.551–1.177) | 0.264 | 0.723 (0.495–1.057) | 0.09 |
| **Pre-hypertension** | | | | | | |
| Nonfatty liver | 1.64 (1.523–1.818) | <0.001 | 1.128 (1.032–1.233) | 0.008 | 1.113 (1.018–1.216) | 0.02 |
| Mild NAFLD | 1.608 (1.424–1.815) | <0.001 | 1.162 (1.027–1.315) | 0.02 | 1.061 (0.937–1.202) | 0.35 |
| Moderate/Severe NAFLD | 1.538 (1.303–1.815) | <0.001 | 1.177 (0.994–1.392) | 0.056 | 1.055 (0.890–1.250) | 0.54 |
| **Hypertension** | | | | | | |
| Nonfatty liver | 3.733 (3.440–4.051) | <0.001 | 1.579 (1.453–1.717) | <0.001 | 1.501 (1.381–1.632) | <0.001 |
| Mild NAFLD | 3.240 (2.958–3.5504) | <0.001 | 1.707 (1.551–1.877) | <0.001 | 1.516 (1.376–1.670) | <0.001 |
| Moderate/Severe NAFLD | 3.346 (3.034–3.691) | <0.001 | 1.836 (1.653–2.039) | <0.001 | 1.544 (1.387–1.720) | <0.001 |

Abbreviations: HR, hazard ratio; NAFLD, non-alcoholic fatty liver disease; BMI, body mass index; FBG, fasting blood glucose; HDL, high-density lipoprotein; LDL, low-density lipoprotein; HsCRP, high-sensitivity C-reactive protein; TG, triglyceride; eGFR, estimated glomerular filtration rate. Model 1 was adjusted for age, sex, physical activity, BMI (≥30, 25–29.9, 18.5–24.9, <18.5), smoke. Model 2 was adjusted for age, sex, physical activity, education, BMI (≥30, 25–29.9, 18.5–24.9, <18.5), smoke, FBG, antidiabetic medication, lipid-lowering medication, TG, LDL, HDL, HsCRP and eGFR.
